# Metabolomics in sturgeon research: a mini-review

**DOI:** 10.1007/s10695-024-01377-8

**Published:** 2024-07-09

**Authors:** Qi Liu, Takeshi Naganuma

**Affiliations:** Graduate School of Integrated Sciences for Life, Hiroshima University, Higashi-Hiroshima, 739-8528 Japan

**Keywords:** *Acipenser*, Omics, Metabolome, Lipidome, Lipidomics, Caviar

## Abstract

Sturgeons are ancient fish, with 27 species distributed in the Northern Hemisphere. This review first touches upon the significance of sturgeons in the context of their biological, ecological, and economic importance, highlighting their status as “living fossils” and the challenges they face in genomic research due to their diverse chromosome numbers. This review then discusses how omics technologies (genomics, transcriptomics, proteomics, and metabolomics) have been used in sturgeon research, which so far has only been done on *Acipenser* species. It focuses on metabolomics as a way to better understand how sturgeons work and how they react to their environment. Specific studies in sturgeon metabolomics are cited, showing how metabolomics has been used to investigate various aspects of sturgeon biology, such as growth, reproduction, stress responses, and nutrition. These studies demonstrate the potential of metabolomics in improving sturgeon aquaculture practices and conservation efforts. Overall, the review suggests that metabolomics, as a relatively new scientific tool, has the potential to enhance our understanding of sturgeon biology and aid in their conservation and sustainable aquaculture, contributing to global food security efforts.

## Introduction

There are 27 species of sturgeons, all with ray-finned bodies but not neopterygian bodies. They are in the family *Acipenseridae*, which is in the class *Actinopterygii*, subclass *Chondrostei*, and order *Acipenseriformes* (Fricke et al. 2023), and has paraphyletic intergenic clades (Luo et al. 2019; Nedoluzhko et al. 2020; Shen et al. 2020). Their natural populations of 25 extant species in the wild (Froese and Pauly 2023; IUCN 2023) are distributed in riverine, lacustrine, estuarine, and coastal waters of the Northern Hemisphere (Pikitch et al. 2005), with non-native populations in South America (Avigliano et al. 2023). They are a remarkable evolutionary relic, earning the designation of “living fossils” by Charles Darwin (1859, p. 107). Positioned at the phylogenetic base of ray-finned fishes, sturgeons, with their archaic forms and ganoid scales, appear “frozen in time” (Du et al. 2020). Teleosts of neopterygian ray-finned fish went through three to four rounds of whole-genome duplications (Pasquier et al. 2016), but sturgeons only went through two rounds, keeping their primitive traits (Cheng et al. 2019; Du et al. 2020; Zhang et al. 2021).

Despite their unique biological characteristics, sturgeons present challenges for genome analyses due to diverse chromosome aneuploidy (Havelka et al. 2011). Studies have found that the number of chromosomes in different sturgeon species varies a lot. They range from 112 for the shovelnose sturgeon (*Scaphirhynchus platorynchus*) (Ohno et al. 1969) to 372 ± 6 for the shortnose sturgeon (*Acipenser brevirostrum*) (Fontana et al. 2008), 437 for the cultured Siberian sturgeon (*A. baerii*) (Havelka et al. 2016), and even 520 for the artificial octoploid Russian sturgeon (*A. gueldenstaedtii*) (Lebeda et al. 2020). These findings make it harder to do genomic engineering. Whole-genome sequencing has been done with only two sturgeon species (*A. ruthenus* and *A. sinensis*) (Cheng et al. 2019; Du et al. 2020; Wang et al. 2023a, b) and one paddlefish (*Polyodon spathula*) of the sturgeon-related family *Polyodontidae* (the order *Acipenseriformes*) (Zhang et al. 2021), besides complete mitochondrial genomes of Atlantic, Gulf, and European sturgeons (*A. oxyrinchus oxyrinchus*, *A. o. desotoi*, and *A. sturio*) (Liu et al. 2017) and an intergenic hybrid (*Huso dauricus* ♀ × *A. schrenckii* ♂) (Popović et al. 2016). Although cytogenetic approaches have already been used in aquaculture and conservation of sturgeons (Chandra and Fopp-Bayat 2021), genomics in sturgeon research has thus only just begun. In contrast, many other aquaculture studies have employed cognitive genomics, comparative genomics, functional genomics, metagenomics, pangenomics, and neurogenomics (Tripathy et al. 2021).

Apart from their biological significance, sturgeons hold economic importance as fishery resources, particularly prized for caviar known as “black gold,” which may not unlikely drive sturgeons to extinction (Boscari et al. 2014). As the need for sturgeon aquaculture grows, problems like slow growth and sexual maturation come up. These problems have led to more quantitative approaches like artificial hatching and feed improvement (Lobanov et al. 2023), as well as genome mining efforts. Genome aneuploidy makes it hard to reproduce artificially, but metabolomics looks like a promising tool that can help build a foundation for sustainable aquaculture of sturgeons. The Convention on International Trade in Endangered Species of Wild Fauna and Flora (CITES) or the Washington Convention (CITES 2001), which states that these fish are ancient survivors who are in danger of going extinct due to human activities, protects them. This mini-review offers insights into metabolomics in sturgeon research, covering its definition, characteristics, methodology, and evolution. It highlights the potential of metabolomics to explore sturgeons’ intricate metabolic systems and understand how external factors impact their internal processes. Additionally, the review discusses the brief history, current status, and perspectives of sturgeon metabolomics, aiming to enhance conservation efforts by improving farmed population breeding.

## The use of omics methodologies in bioscience research

The rapid progress in bioscience research underscores the need for multifaceted approaches to fully grasp organismal growth and evolution. Contemporary scientists are engaged in comprehensive investigations into various aspects of organisms’ lives, leading to the emergence of “omics” technology. Omics facilitates systematic exploration of biological processes, encompassing entities such as the genome, transcriptome, proteome, and metabolome. Its objective is to identify, describe, and quantify biomolecules involved in fundamental biological activities like metabolism and energy production (Vailati-Riboni et al. 2017).

**Fig. 1:**
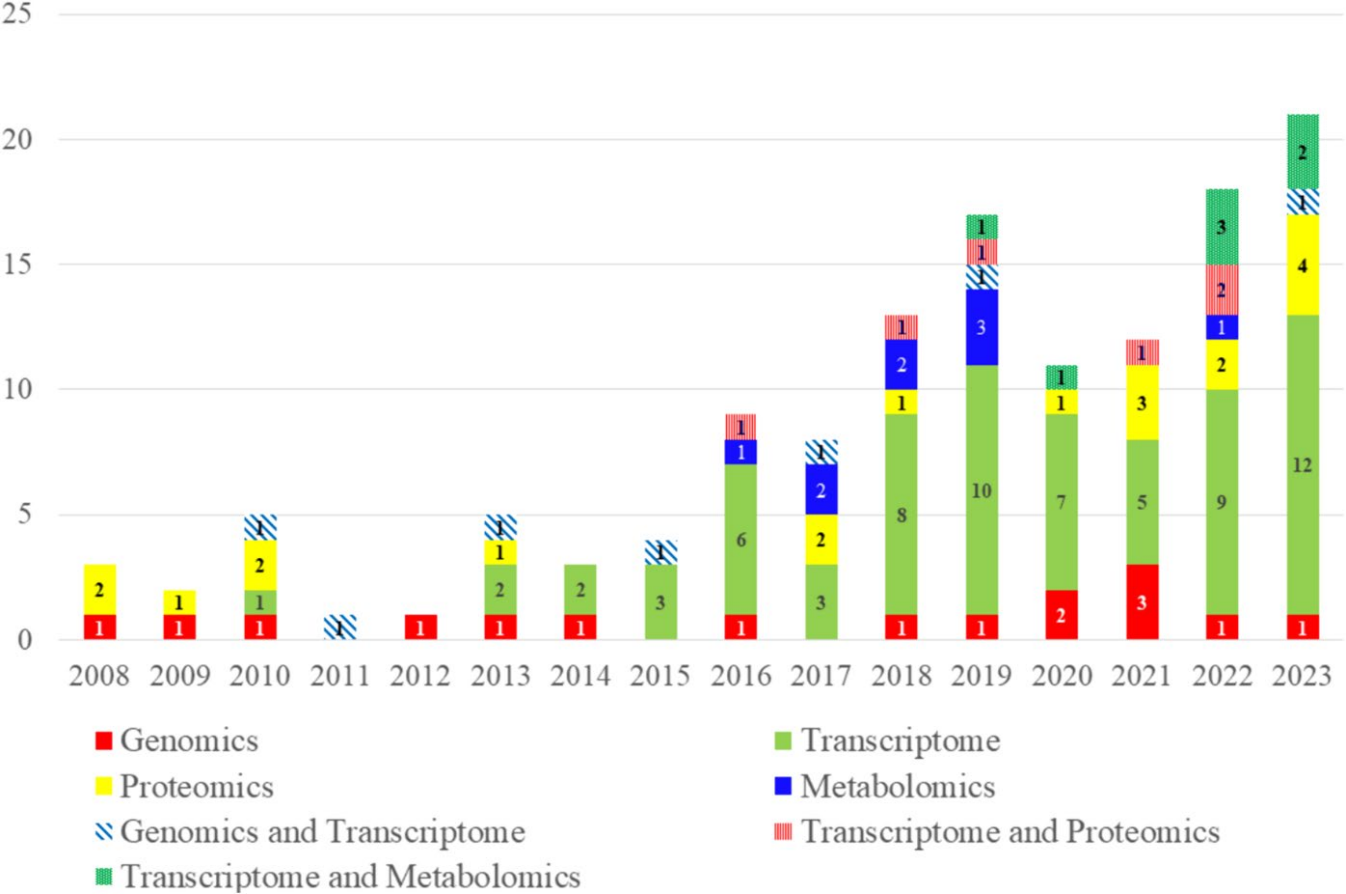
Numbers per year of the publications on omics applications to sturgeon studies. Metabolomics-related publications (nine “Metabolomics” and seven “Transcriptomics and Metabolomics”) have appeared since 2016

Omics technologies such as genomics, transcriptomics, proteomics, and metabolomics have advanced rapidly, enabling precise analysis of complex biological systems. Applying these technologies to sturgeon research can enhance our understanding of this endangered species’ life processes and aid in its conservation. This review highlights the use of metabolomics and other omics approaches in sturgeon studies (Fig. 1).

## Application of genomics, transcriptomics, and proteomics in aquaculture research

The world is facing a food crisis due to climate change, societal development, and geopolitical issues, with a growing demand for animal protein. Fisheries resources are essential providers of proteins and nutrients, supporting the livelihoods of 200 million people—a number expected to rise. However, overfishing and habitat degradation are depleting wild fishery resources, making aquaculture the primary method of obtaining these resources. Since 2000, aquaculture has significantly grown in the global food chain, with freshwater aquaculture predominant in Asia and Africa, and marine aquaculture in Europe, the Americas, and Oceania (Verdegem et al. 2023).

Aquaculture requires strict criteria, including temperature, microbial communities, feed, water quality, environment, and reproduction. Enhancing these conditions promotes sustainable growth. Advances in omics technologies—transcriptomics, proteomics, metabolomics, and genomics—provide new research methods for aquaculture. Huete-Pérez and Quezada (2013) highlighted genomics’ role in studying traits, sex identification, genetic structure, and environmental responses. Qian et al. (2013) reviewed transcriptomics, which helps understand stress responses to osmotic pressure, pathogens, and temperature changes, as well as mechanisms of early development and population differentiation. Zhou et al. (2012) reviewed proteomics in aquaculture.

Proteomics technology is crucial for studying fish resource dynamics, physiological adaptation, and biodiversity, assessing the impact of feed composition changes, and ensuring seafood safety and quality. Metabolomics has further advanced aquaculture by providing metabolite profiles that reflect an organism’s phenotype and environmental sensitivity, offering insights into breeding conditions and improving breeding systems. Alfaro and Young (2018) highlighted metabolomics’ significance in aquaculture, examining its application in nutrition and food, hatchery production, post-harvest quality control, disease, and immunology.

We searched for literatures related to sturgeon proteomics and a total of 19 literatures were searched. Keyvanshokooh et al. (2009a, b) used proteomics techniques to reveal the effects of dietary methylmercury on proteome modifications in the brain of juvenile beluga (*Huso huso*). The results of differential protein profile expression indicated that the toxic mechanism of methylmercury is derived from oxidative stress induction and apoptotic effects and that chronic exposure to methylmercury leads to severe metabolic defects in the brain. This study reveals the adverse effects of hazardous substances on sturgeon species, which will guide sturgeon conservation and improvement of the living environment. Murphy et al. (2020) used proteomics to study changes in protein profiles in the epidermal mucus of Atlantic sturgeon under external environmental stress as a means of identifying potential protein biomarkers of stress. The present study provides a novel and non-invasive measurement to understand the response mechanisms of Atlantic sturgeon when subjected to environmental stress, which will contribute to the improvement of the Atlantic sturgeon’s survival environment. Silvestre et al. (2010) used proteomics analysis to investigate the differential expression of proteins in green sturgeon and white sturgeon larvae under heat stress and selenium stress, and the differential proteins can serve as biomarkers for environmental stressors in sturgeon fingerlings. The discovery of biomarkers in this study will contribute to a better assessment of sturgeon health and provide new research tools for monitoring the sturgeon’s survival environment. This will greatly contribute to sturgeon species conservation. Salimi Khorshidi et al. (2021) assessed the effects of bisphenol A on Siberian sturgeons (*Acipenser baerii*) using proteomic techniques. The proteomic results showed that bisphenol A increased the expression of proteins involved in detoxification and metabolism, activated glycolysis, and led to hepatic necrosis, which will provide insights into the conservation of the sturgeon species and improvement of the living environment.

Liu et al. (2022) used a label-free quantitative proteomics approach to reveal the molecular mechanism of off-flavor produced by low-temperature vacuum heating (LTVH)-treated Russian sturgeon (*Acipenser gueldenstaedti*) fillets. A total of 120 flavor-related proteins were identified. Hexanal, heptanal, nonanal, and octanal were identified as the sources of off-flavor in LTVH-treated sturgeon, confirming the association of protein oxidation with off-flavor and suggesting a potential link between biological processes and the formation of off-flavor. Another study from Shen et al. (2022) also revealed the molecular mechanism by which the texture of Russian sturgeon (*Acipenser gueldenstaedtii*) fillets was improved by the low-temperature vacuum heating technique. Similarly, Cai et al. (2021) used comparative proteomics to investigate the preservation effect of low-temperature vacuum cooking on Russian sturgeon (*Acipenser gueldenstaedti*) meat, and the results showed that low-temperature vacuum cooking was able to limit the growth of harmful microorganisms while at the same time causing lipid oxidation and oxidative degradation of proteins. Solving the problem of protein oxidation will be a prerequisite for the application of low-temperature vacuum cooking. Jiang et al. (2023) used proteomics technology to explore the mechanism of the effect of low-temperature vacuum heating on the processing quality of sturgeon meat from the perspective of protein oxidation. A total of 733 proteins were identified. Through the oxidation site delineation, it was determined that low-temperature vacuum heating-induced protein oxidation was caused by malondialdehyde and 4-hydroxynonenal. The results of the study can provide a theoretical basis for the precise processing of aquatic products.

Boccaletto et al. (2018) summarized the application of various proteomics techniques in studies related to the study of the protein composition of fish spermatozoa and other studies with a view to using proteomics techniques to investigate the mechanisms of post-testicular maturation in sturgeon. Kodzik et al. (2023) used proteomics techniques to parse the seminal plasma proteome of Siberian sturgeon and to analyze the functions of the identified proteins. This study provides deeper insights into the physiological functions of seminal plasma and the processes occurring in the sturgeon reproductive tract. The sturgeon seminal plasma proteome opens the way for future research to develop new biomarkers and possible applications to improve controlled reproduction methods. Kodzik et al. (2024) also performed a comparative proteomic analysis of Siberian sturgeon ovarian fluid and eggs to comprehensively characterize the protein composition of Siberian sturgeon ovarian fluid and eggs and further analyze the functions, roles, and metabolic pathways of the differential proteins. Li et al. (2017) used proteomics techniques to resolve and compare the proteomes of spermatozoa from two sturgeon species (*Acipenser baerii* and *A. schrenckii*) to assess sperm quality, while comparative proteomic analyses of high-quality spermatozoa versus low-quality spermatozoa showed that sperm quality was correlated with the expression levels of certain proteins. The results of this study will further elucidate the potential role of proteins in sturgeon spermatozoa, which will be of great help in the reproductive management of endangered species. Similarly, Li et al. (2010) used comparative proteomics techniques to comparatively analyze the protein profiles of spermatozoa from six sturgeon species, and the identification results suggest that differential proteins or protein isoforms can be used to distinguish sturgeon species. This study refines the construction of a sturgeon protein database, points to the application of proteomics for differential characterization and comparative studies of sturgeon species at the molecular level, and will provide a valuable resource for understanding the molecular mechanisms of spermatogenesis and spermatogenesis in sturgeon. Shaliutina et al. (2013) used comparative proteomics to evaluate *Acipenser ruthenus* seminal plasma and analyze the effect of multiple sperm collections on protein composition. The results showed that although multiple semen collections do not significantly alter sperm quality, they do alter the protein pattern of seminal plasma. This study refines the molecular mechanisms underlying the protective role of the protein composition of seminal plasma for spermatozoa during storage in the reproductive system. It provides a theoretical basis for the artificial breeding of sturgeon. Niksirat et al. (2017) explored the changes in the egg proteome during fertilization of sterlet *Acipenser ruthenus* using proteomics techniques and showed that in vitro fertilization of sturgeon eggs is accompanied by the release of a large number of different proteins into the external environment and that these proteins may be involved in constructing a temporary microenvironment around the egg that attracts and protects spermatozoa and ensures subsequent fertilization.

Keyvanshokooh and Vaziri (2008) analyzed the proteomic profile of Persian sturgeon (*Acipenser persicus*) ova using proteomics techniques to determine their protein composition. The sturgeon ova protein database was supplemented while providing a resource for molecular analysis to understand the reproductive status of female sturgeon. The resolution of the protein composition of sturgeon ova also provides a better understanding of the factors affecting the quality of sturgeon caviar during refrigeration. Keyvanshokooh et al. (2009a, b) used a proteomic approach to analyze the differential expression of proteins in the gonads of mature male and female Persian sturgeon (*Acipenser persicus*). The differentially expressed proteins were mainly found in the testis. The identified differentially expressed proteins were mainly involved in life processes such as metabolism, transcription, and translation, and none of the differentially expressed proteins was associated with sex-determining genes. Youneszadeh-Fashalami et al. (2018) used proteomic techniques to compare the characteristics of the proteomics of the ovarian vitellogenesis stage of the sturgeon (*Acipenser ruthenus*). Protein expression pattern changes from defense to metabolism. This study refines the molecular mechanism of oocyte maturation in sturgeon (*Acipenser ruthenus*). Lv et al. (2021) identified important proteins involved in gonadal development in the gonads and pituitary gland of *Acipenser schrenckii* under the regulation of gonadotropin-releasing hormone—a treatment using proteomics technology. These differentially expressed proteins play crucial roles in signal transduction and endocrine metabolic pathways. This provides a resource for understanding the mechanisms and processes of sturgeon gonadal development, as well as a theoretical basis for sturgeon aquaculture. Among the 24 studies on sturgeon genomics, six focused on sex identification (Keyvanshokooh and Gharaei 2010; Burcea et al. 2018; Lasalle et al. 2021; Ruan et al. 2021; Degani et al. 2022; Panagiotopoulou et al. 2023). Eleven studies examined sturgeon population structure, phylogeny, evolution, and species identification (Sato and Nishida 2010; Doukakis et al. 2012; Ogden et al. 2013; Albayrak et al. 2014; Boscari et al. 2015, 2017, 2021; Trifonov et al. 2016; Shen et al. 2020; Whitaker et al. 2020; Stundl et al. 2023). Four studies investigated sturgeon immunogenomics, aiming to understand and improve immune-related genes (Mugue et al. 2019; Tang et al. 2019; Höhne et al. 2021; Soto et al. 2021). Three studies improved sturgeon genome data (Lazzari et al. 2008; Viegas et al. 2008; Ma et al. 2011).

Sturgeon genomics research mainly focuses on identifying sturgeon traits and improving genomic information, which can guide sex and species identification. However, artificial breeding and aquaculture are crucial to preventing sturgeon extinction. Genomic research, which explores genetic mechanisms, cannot directly observe the impact of external pressures (e.g., temperature, osmotic pressure, dissolved oxygen, feed, disease prevention, water quality, microorganisms) on sturgeon growth and metabolism. Metabolomics can more clearly demonstrate sturgeons’ growth laws and response mechanisms and accurately reflect their physiological state, playing a significant role in addressing aquaculture issues and improving conditions. Current genomics research on sturgeons remains at the stage of enhancing genomic information and identifying traits, which supports aquaculture development, but metabolomics offers clearer insights into sturgeon physiology and better solutions for aquaculture challenges.

## Metabolomics

Metabolomics, a relatively new field introduced by Professor Jeremy Nicholson of Imperial College London in the mid-1990s, emerged from the investigation of the metabolome. The term “metabolome” refers to all small molecules with a molecular weight less than 1500 Da present during a specific physiological period in a cell, organ, tissue, organism, or non-living things like food and feed (Qiu et al. 2023). Metabolomics involves the qualitative and quantitative, or semi-quantitative, study of these small molecules to examine metabolic pathways and link metabolome changes to physiological processes in living organisms.

Metabolomics analysis technology is valuable in nutritional research as it reveals how external substances affect an organism’s internal processes, such as growth, development, metabolism, and reproduction. As a relatively new scientific tool, metabolomics builds on advances in genomics, transcriptomics, and proteomics to study complex metabolic processes in organisms. Unlike other omics fields, which focus on gene expression, metabolomics studies the end products of these processes, the metabolites generated by metabolic pathways (Jacob et al. 2019).

The results of metabolomics research are closely related to the phenotype of organisms. Fiehn (2002) noted that this research helps us understand how organisms change and respond to environmental stimuli, accurately describing their physiological states. Subtle differences in genome and proteome expression can translate into metabolite differences, making them easier to identify. Unlike genomics and transcriptomics, which require high-throughput sequencing and large databases, metabolome analysis is more intricate yet simpler. Metabolites have significantly lower molecular masses than genes and proteins and are independent of genetics or species (Taylor et al. 2002).

## Application of metabolomics in sturgeon research

Sturgeons are endangered species but important fishery resources in Eurasia and North America. Biotechnology, including omics, has gradually revealed biological characteristics such as the genetic structure and physiological attributes of sturgeons, promoting the development of the sturgeon breeding industry. Compared with genomics, transcriptomics, and metabolomics, there are few reports on sturgeon metabolomics. With “sturgeon” and “metabolomics” as keywords, 16 articles related to sturgeon metabolomics were searched in the Web of Science database. The studies have been limited to the *Acipencer* species, and none has been published for the other three genera (*Huso*, *Pseudoscaphirhynchus*, and *Scaphirhynchus*).

Zhou et al. (2023) studied the energy sources and patterns of use during the embryonic development of the Chinese sturgeon. They did this by looking at the biochemical make-ups of embryos at different stages of development and combining this with transcriptomics to learn more about the embryos’ physiological traits and the molecular processes involved during embryogenesis. The changes in protein and lipid content and the differences in amino acid and fatty acid content in embryos at different developmental stages can objectively reflect the utilization of lipids and proteins as energy sources during embryonic development. Compared with genomics and transcriptomics, changes in protein and lipid content more clearly and directly reflect the metabolic profiles of proteins and lipids. The protein and lipid metabolic maps intuitively reflect the energy sources during embryonic development. Changes in the biochemical make-up of the fish show that the lipids provided the energy substrates used in the early stages of embryonic development.

In contrast, the proteins provided the energy needed for late embryonic development, differentiation, and metabolism. Transcriptomics analysis was used to look at the genes and pathways that were differentially expressed and how they affected growth and nutrient metabolism in embryos at different stages of development. The results showed that factors related to protein processing were highly upregulated in the early embryonic development stage, and the energy for protein processing may come from lipid metabolism. The study of changes in transcriptomes and changes in biochemical composition gives us a theoretical foundation for a first understanding of how embryonic development works physiologically and at the molecular level. The analysis of biochemical composition in this study was limited to the analysis of protein and lipid macromolecules. The comprehensive analysis of small-molecule metabolites can more comprehensively interpret the energy metabolism profile. The results of this research provide certain data to support the study of the early embryonic development of sturgeons. The good development of early embryos of sturgeon is the basis of sturgeon breeding, which will contribute to the breeding of Chinese sturgeon and the output of high-quality caviar.

Zhu et al. (2020) studied changes in lipid and amino acid metabolism and how they work energetically during ovarian development in Chinese sturgeons. They looked at serum metabolomes and gene expressions in the ovary. The results showed that the metabolisms of most lipids and amino acids are active during ovarian development stages II and III. In contrast, the contents of histidine, alanine, and sarcosine, as well as most fatty acids and their derivatives, peak during ovarian development stage IV. The energy required for ovarian development comes from two sources: exogenous food sources and endogenous sources. From stage II to stage III of ovarian development, a large amount of exogenous lipids is absorbed into the ovaries and oxidized for energy. This finding indicates that the energy required for ovarian development mainly comes from lipid and amino acid metabolism. Another study by Du et al. (2019) used serum metabolomics and gonad transcriptomics to show how dietary lipid levels can help the development of ovaries in Chinese sturgeons.

The results showed that diets with high lipid content favor synthesizing steroid hormones, cholesterol, yolk production, and arachidonic acid metabolism. Differently expressed genes and metabolites point to this conclusion. However, it needs to be confirmed by measuring enzyme activity and looking at the differences in the rates of making cholesterol and fatty acids. In short, lipid metabolism is the main source of energy for ovarian development. The high-lipid diet promotes the development of gonads in female Chinese sturgeons. The metabolite profiles in the ovaries and liver can more intuitively reflect the metabolic differences in different stages of ovarian development. However, the metabolomics analysis samples from these two studies were limited to serum. Subsequent ovarian metabolism-related studies should use ovarian samples or liver samples so as to accurately and objectively explain the metabolic changes in different stages of ovarian development.

Rahimi et al. (2019) used ^1^H NMR-based metabolomics to look into what changed in Persian sturgeon’s sperm metabolites after being frozen in straw (pipette) with 2-hydroxypropyl-beta-cyclodextrin (H^2^CD) as an outside cryoprotectant. By adding 10 mM H^2^CD, some metabolites were successfully preserved, including glucose, guanidinoacetate, O-phosphocholine, and N, N-dimethylglycine. Other metabolites, including lactate, carnitine, betaine, β-alanine, and trimethylamine N-oxide, were not as well preserved. This finding helps develop the use of HCD as a cryoprotectant. HCD, a cryoprotectant, cannot stop the freezing and thawing processes for metabolites that are highly temperature-sensitive, particularly creatine phosphate and creatine as energy resources.

Abed-Elmdoust et al. (2017) also used ^1^H NMR to study the chemicals in the sperm of a wild Persian sturgeon that had been preserved with type III fish antifreeze protein (AFP). The semen was droplet-vitrified in liquid nitrogen, not straw-cryopreserved. The results showed that the AFP used was effective as a cryoprotectant, as shown in another of their articles from the metabolite perspective. Abed-Elmdoust et al. (2019) used metabolomics to compare “droplet vitrification” with the common straw cryopreservation of Persian sturgeon. It was found that droplet vitrification is better at keeping metabolites that are important for sperm energy metabolism, redox homeostasis, and compensating for low oxygen levels. These three studies on the transformation and comparison of sturgeon sperm cryopreservation technology have always been unable to avoid the effective preservation of creatine phosphate and creatine as energy resources. The frozen metabolomics analysis samples came from sperm samples that had been used to check the quality of the sperm. During the semen quality check, the sperm will consume some metabolites, which could lead to differences in the experimental results. Sturgeons are rare protected animals and face the risk of extinction. Therefore, the preservation of sturgeon sperm is of great significance to the protection of sturgeon species, and it also plays a positive role in the large-scale artificial breeding of sturgeon aquaculture. The study of how to cryopreserve sturgeon sperm is very important because of this, and the use of metabolomics research methods has expanded the study of sperm protection mechanisms.

As a rare species, the protection and artificial breeding of sturgeons are the key to solving the endangered species problem. The study of the ovary, embryo, and sperm preservation technology can theoretically reveal the reproduction and growth mechanism of sturgeons. The application of metabolomics as an emerging omics technology in sturgeon-related research will enable us to have a more comprehensive understanding of the mechanism of sturgeon reproduction and growth. It will also make great contributions to the protection, artificial breeding, and aquaculture of sturgeons.

Li et al. (2022) performed a non-biological but culinary metabolomics study that revealed the effects of heat treatment on the flavor and sensory characteristics of the meat of juvenile hybrid sturgeon (*Acipenser gueldenstaedtii* ♀ × *A. schrenckii* ♂). They showed that steaming for 12 min leads to optimum sensory quality and flavor stability and that phosphatidylethanolamine bound with unsaturated fatty acids 18:2, 20:4, and 22:6 is the key flavor precursor. Sensory attributes and flavor evaluation are described and evaluated by members of the sensory panel. Hence, the description of flavor characteristics is likely to be affected by personal factors and is more subjective. The use of metabolomics methods to identify volatile components and non-targeted metabolites can objectively and directly reflect the differences in volatile substances and metabolites of sturgeon under different temperature treatments. The difference in metabolites intuitively shows the different sources of flavor. This study is on the post-processing technology of sturgeon aquaculture, which provides a theoretical basis for the post-processing mode of sturgeon meat, which will also promote the development of sturgeon farming.

Heude et al. (2016) used ^1^H NMR spectroscopic metabolomics to characterize the metabolites of caviar and differentiate the caviars of Russian and Siberian sturgeons, *Acipenser gueldenstaedtii* and *A. baerii*, respectively, from five breeding sites. Metabolomic profiles of the caviars may be used to prevent counterfeiting at renowned caviar production sites and to guarantee adequate freshness. Caviar can be traced back through identification methods such as mitochondrial DNA or microsatellite DNA analysis. However, for the caviar production industry, the quality of caviar varies greatly from species to species in different production areas and under different breeding conditions. Therefore, the genetic identification method for identifying caviar from high-quality production areas could be more rigorous. This study used the analysis of differences in the metabolomics characteristics of caviar to provide new ideas for the identification of caviar production areas. The distribution of caviar metabolites is affected by various factors. Therefore, when commercially identifying the origin of caviar, a large number of samples are needed to establish the model, and the sample model needs to be updated regularly.

Sturgeon meat can be used as a high-quality protein source on the table, and caviar is an expensive delicacy on the table. The quality, flavor, and nutrients of food depend on the various organic and inorganic components in the food. Therefore, the metabolomics analysis of sturgeon meat and caviar can fully explain why they can be used as high-quality food. A comprehensive analysis of sturgeon as a high-quality food can promote the development of sturgeon aquaculture.

Wu et al. (2022) investigated the possible mechanism of arachidonic acid, 20:4(ω-6), as a feed supplement to promote steroid hormone synthesis in the female Chinese sturgeon (*Acipenser sinensis*) ovary by transcriptomics and lipidomics, i.e., lipidic metabolomics (Wenk 2005). Wu et al. (2022) objectively demonstrated the differentially expressed genes and metabolites of Chinese sturgeons fed with feeds containing different arachidonic acid contents (0%, 0.5%, 1%, and 2%). The result showed that arachidonic acid regulates steroid synthesis by enhancing cholesteryl ester metabolism and promoting the transcription of genes for steroidogenesis. The modulation of gonadal development and cholesterol synthesis would promote ovary development in the offspring of Chinese sturgeon. In this study, the difference in cholesterol ester content in samples of different groups was speculated to be caused by the content of arachidonic acid. However, related enzymes strictly regulate the synthesis and hydrolysis of cholesterol ester in the body. If the quantitative analysis of cholesterol esterase and cholesterol ester hydrolase can be increased, it will further support this speculation. This study supplemented the research content on the effect of feed additives on the development of sturgeon gonads, provided a certain theoretical basis for the study of sturgeon gonad development, and also provided help for the reproduction of sturgeon in sturgeon aquaculture.

Feed is the most important part of aquaculture. Various ingredients in feed can affect the growth, development, and health of sturgeons. Fishmeal is the main source of protein in feed. Due to the cost of breeding, soybean meal appears in the feed market as a substitute for fish meal, but excessive soybean meal will affect the healthy growth of fish. In order to eliminate the adverse effects of soybean meal as a necessary ingredient in feed on the growth of sturgeons, Yue et al. (2022) investigated the effects of glutamine as a soybean meal dietary additive on the growth and liver metabolism of hybrid sturgeon (*Acipenser baerii* ♀ × *A. schrencki* ♂) using transcriptomics and metabolomics techniques. The changes in physiological indicators of hybrid sturgeon (*Acipenser baerii* ♀ × *A. schrencki* ♂) objectively showed that glutamine supplementation could reduce the negative effects of soybean meal-induced liver damage, growth retardation, and alteration of proximal muscle composition. The ratio of feed ingredients in the experimental design of this study caused an increase in potential variables. However, the potential variables did not dominate the nutritional composition and would not have a significant impact on the growth of the body. As a conditionally essential amino acid, the specific molecular mechanism by which glutamine plays a protective role as a feed additive needs further study. In addition to the growth effect, sturgeon breeding is an extremely important part of sturgeon aquaculture, and the effect of glutamine as a feed additive on sturgeon breeding also needs to be studied. Similar to this study, Wang et al. (2023a, b) explored the negative effects on the growth and physiology of Amur sturgeon caused by the replacement of fishmeal with cottonseed protein concentrate in mixed feeds containing soybean, wheat, and fish oil. The replacement resulted in reduced weight gain efficiency, feed efficiency, and essential amino acid content and decreased digestive enzyme activity of the tested sturgeon. Different from the results in the study by Yue et al. (2022), soybean meal as a fish meal substitute led to an increase in the concentration of multiple amino acids. In general, the selection of fish meal substitutes is the development trend of aquaculture feeds, and eliminating the negative effects of substitutes on the body is also a problem that must be solved. The above two studies provide help for the development of feed in aquaculture and provide ideas for the sustainable development of aquaculture.

Feed is the most important part of sturgeon aquaculture, and it is the main source of food and energy. The cost of feed will affect the profit of aquaculture. Similarly, the benefits of sturgeon aquaculture also depend on the quality of sturgeons, and the composition of feed will affect the quality of sturgeons by affecting their growth and metabolism. Therefore, the selection of the best feed and the study of the effect of feed on the physiological metabolism of sturgeons will greatly affect the development of sturgeon aquaculture.

Lin et al. (2019) explored the metabolic effects of feed restriction on green sturgeon (*Acipenser medirostris*) fingerlings using a metabolomics approach. Feed restriction is a simulation of food shortage in the natural environment. Biologically, food shortage will have an impact on the population of wild animals. Metabolic studies under feed restriction can reflect the physiological changes of wild sturgeons under environmental stress. The results indicated that feed restriction affects physiological processes such as osmotic pressure regulation, energy metabolism, and antioxidants in sturgeon. Among the extracted metabolites from sturgeon meat, liver, and kidney, hydrophilic metabolites responded more to feed restriction than hydrophobic metabolites, and kidney tissue responded more strongly than liver and muscle among different tissues. Most amino acids and most fatty acids decreased, while creatine phosphate, taurine, and glycine increased significantly under feed restriction. The timing of dietary restriction in this study deserves further consideration. Sturgeons will experience a short-term stress response under short-term dietary restrictions. However, in the face of long-term dietary shortages, sturgeons will gradually produce physiological adaptations and tend to a steady state. The optimal feeding rates vary between species, and the optimal feeding rates of the same species at different body sizes may also vary. The reference to the optimal feeding rate of the white sturgeon in this study may cause deviations in the experimental results. Overall, this study outlines the negative effects of feed restriction on the physiological metabolism of *Acipenser medirostris*, which provides a new idea for studying the physiological and functional changes of wild animals under environmental stress.

Environmental changes caused by human activities have seriously affected the reproduction of sturgeon species. Human noise is the main source of noise in the aquatic environment. Noise seriously affects the growth and development of fish and the physiological growth process. Noise factors may also be one of the factors that cause sturgeon populations to face the risk of extinction. Zhang et al. (2022) investigated the mechanism of liver metabolism in hybrid sturgeon (*Acipenser baerii* ♀ × *A. schrencki* ♂) as the stress responses from ship noise using transcriptomics and metabolomics techniques. This study selected liver tissue as the experimental sample because the liver is the metabolic center of the body, and liver-level omics research is a microcosm of body omics research. The results of transcriptomics and metabolomics tests objectively and clearly show the differences between the transcriptome and metabolome of sturgeon before and after the influence of noise. The different results showed that noise exacerbated apoptosis and motility in sturgeon and inhibited DNA replication, RNA transcription, and protein synthesis. In terms of metabolism, the metabolic pathways of lipids, nucleotides, and vitamin D3 were also inhibited. These objectively demonstrate the adverse effects of noise pollution on the physiological processes of sturgeons. In terms of energy metabolism, the hybrid sturgeon met the material and energy requirements under noise stress by enhancing carbohydrate and amino acid metabolism. This study is the first to reveal the adverse effects of water environment noise on the growth process of hybrid sturgeon. The results of this study can play a certain role in evaluating the impact of underwater noise on fish in aquaculture and, at the same time, guide the improvement of hybrid sturgeon breeding conditions. Through transcriptome and metabolome analysis, sturgeon breeding conditions can be optimized to achieve the best breeding conditions for sturgeon.

Hajirezaee et al. (2017) used ^1^H-NMR-based metabolomics to investigate the metabolic effects of Persian sturgeon (*Acipenser persicus*) fingerlings adaptation to brackish water (12% sea salts) and exposure to sub-lethal levels of diazinon, an organophosphate insecticide. The results indicated that diazinon activates the gluconeogenesis processes to generate energetic metabolites such as acetate, acetoacetate, and glycerol. Diazinon thus influences the osmoregulatory processes of Persian sturgeon. However, in terms of experimental design, as the diazinon is naturally degraded and the salinity of the water body is changed, the water environment naturally transitions from a toxic water environment to a hypertonic non-toxic water environment, which creates potential variables for the subsequent saltwater adaptation experiment. Hajirezaee et al. (2018) also explored the metabolic adaptation and osmoregulation of the endangered Persian sturgeon fingerlings to brackish water salinity (1.2% sea salt). They showed that certain plasma osmolytes and amino acids decline during acclimation to brackish water. Still, the levels of plasma glycine and glucose elevate in brackish water, implying glycolysis contributes more to the energetic requirements of osmoregulation than lipid oxidation. In contrast, the experimental variable in this study was only water salinity, which can more objectively reflect the metabolic adaptation and osmotic adaptation of Persian sturgeon to salt water. The two studies analyzed the osmotic regulation and water environment adaptation of sturgeon from a metabolic perspective, providing a theoretical basis for the artificial breeding and aquaculture of wild sturgeon.

The water environment is the environment that fish depend on for survival. In recent years, global warming has caused changes in the living environment of fish, and the survival of high-temperature sensitive fish is facing environmental stress. In this context, Chen et al. (2023) conducted a comprehensive analysis of *A. dabryanus* under heat stress by combining pathology, transcriptomics, and metabolomics (UPLC-MS/MS). The results showed that *A. dabryanus* was damaged in intestinal and liver tissues under heat stress. In terms of genes, *A. dabryanus* can achieve heat resistance through the upregulation of heat shock protein genes and some immune genes. In terms of metabolism, amino acid metabolism activities and glucose metabolism activities were significantly enhanced, while lipid metabolism was inhibited. This study has improved the physiological processes and stress mechanisms of sturgeon species under different environmental stresses, which will provide a theoretical basis for the detection and breeding of sturgeon and the protection of wild species under the background of global warming.

Environmental stress is a key factor affecting the growth and survival of wild sturgeon species. The aquaculture environment also affects the growth and metabolism of sturgeons. The study of the impact of environmental stress on sturgeons has guiding significance for the protection of wild sturgeons. Also, it provides a theoretical basis for the optimization of the sturgeon aquaculture environment. The advantages of metabolomics make the physiological and metabolic changes of sturgeons under environmental stress more intuitive. The application of metabolomics makes the study of the impact of environmental stress on sturgeon growth more comprehensive and intuitive.

Sixteen comprehensive reports on sturgeon metabolomics highlight the extensive research in this area. Two studies focused on sturgeon nutrients, three on the effects of sturgeon feed, five on environmental stress responses, and six on gonadal/embryonic development and sperm cryopreservation. Wild sturgeon populations are declining due to overfishing and habitat destruction, leading to extinction risks. However, the demand for sturgeons in aquaculture is increasing due to their high-quality protein and caviar. Future research will focus on artificial breeding and aquaculture, which require strict conditions for breeding and the environment. Improving these conditions will enhance the efficiency and sustainability of sturgeon aquaculture.

Metabolomics aids in understanding sturgeon metabolic processes, improving aquaculture conditions, and enhancing industry sustainability. It identifies key metabolite markers and pathways affected by nutrients to enhance feed nutrition. Metabolomics is crucial for disease management, identifying biomarkers for sturgeon diseases. It evaluates the effects of environmental changes on sturgeon growth and aquaculture systems. Metabolomics also helps in selective breeding programs by identifying metabolites associated with desired traits like growth rate and disease resistance.

In summary, with the rapid development and intervention of metabolomics, human beings will have a more comprehensive and in-depth understanding of sturgeon, not only *Acipenser* but also *Huso*, *Pseudoscaphirhynchus*, and *Scaphirhynchus* species, to help in population conservation and better aquaculture practices.

## Future perspectives of metabolomics in sturgeon research

Aquaculture is one of the main ways the world’s population increases food security. Aquaculture development is determined by animal health, nutritional enhancement, meat quality, and sex identification. Metabolomics advances our knowledge of aquatic creatures’ metabolic processes, enhances aquaculture methods, and increases the sector’s sustainability. Regarding nutritional diets, metabolite profile analysis helps optimize feed formulation by screening important metabolic indicators and pathways that certain nutrients impact (Roques et al. 2020). Regarding disease, metabolomics is essential for locating biomarkers linked to illness in aquaculture species (Kuo et al. 2022).

Metabolomics is a tool that can be used in environmental detection to evaluate the effects of changes in various environmental variables, such as temperature, osmolality, water quality, and environmental pollutants (Koyama et al. 2015). Metabolomics can illuminate an organism’s physiological reactions by identifying pertinent metabolic markers and pathways from outside stimuli (Lulijwa et al. 2022). In order to improve the nutrition and quality of aquaculture goods, metabolomics is utilized to investigate the metabolites that give aquaculture products their flavor and aroma (Diez-Simon et al. 2019). Metabolomics supports selective breeding techniques by identifying metabolites linked to desired growth rate, disease resistance, and stress tolerance (Meng et al. 2023). Metabolomics is continually developing and improving, and its uses in aquaculture are expanding thanks to the creation of metabolite detection platforms and advanced data processing tools.

In summary, metabolomics not only has much promise but also has obstacles that must be gradually solved. The current path of metabolite detection platform development is toward creating numerous platforms to get complete metabolite profiles, as the sensitivity and coverage of existing platforms have significant limits. The application field of metabolomics will be substantially expanded by developing data processing and analysis tools for downscaling and transforming massive amounts of complicated metabolomics data, which are high-dimensional. Implementing standardized schemes, data sharing, quality control, and other initiatives will gradually resolve the problems with repeatability and standardization of metabolomics data. Future metabolomics application domains will expand as research continues to deepen and as big data and artificial intelligence progress, hence gradually solving the problems.

## Data Availability

Data availability is not applicable to this article as no new data were created or analyzed in this study.
